# Prognostic Value of Estimated Glucose Disposal Rate in Patients with Non-ST-Segment Elevation Acute Coronary Syndromes Undergoing Percutaneous Coronary Intervention

**DOI:** 10.31083/j.rcm2401002

**Published:** 2023-01-03

**Authors:** Chi Liu, Qi Zhao, Xiaoteng Ma, Yujing Cheng, Yan Sun, Dai Zhang, Yujie Zhou, Xiaoli Liu

**Affiliations:** ^1^Department of Cardiology, Beijing Anzhen Hospital, Beijing Institute of Heart Lung and Blood Vessel Disease, Beijing Key Laboratory of Precision Medicine of Coronary Atherosclerotic Disease, Clinical Center for Coronary Heart Disease, Capital Medical University, 100029 Beijing, China; ^2^Department of Cardiology, Cardiovascular Center, Beijing Friendship Hospital, Capital Medical University, 100052 Beijing, China

**Keywords:** estimated glucose disposal rate, non-ST-segment elevation acute coronary syndrome, percutaneous coronary intervention, prognosis

## Abstract

**Background:**

Estimated glucose disposal rate (eGDR) is highly associated with all-cause mortality in type 2 diabetes mellitus (T2DM) cases undergoing coronary artery bypass grafting (CABG). Nevertheless, eGDR’s prognostic value in non-ST-segment elevation acute coronary syndrome (NSTE-ACS) following percutaneous coronary intervention (PCI) remains unknown.

**Methods:**

The population of this retrospective cohort study comprised NSTE-ACS patients administered PCI in Beijing Anzhen Hospital between January and December 2015. The primary endpoint was major adverse cardiac and cerebral events (MACCEs). eGDR was calculated based on waist circumference (WC) (${\mathrm{eGDR}}_{\text{WC}}$) or body mass index (BMI) (${\mathrm{eGDR}}_{\text{BMI}}$).

**Results:**

Totally 2308 participants were included, and the mean follow-up time was 41.06 months. The incidence of MACCEs was markedly increased with decreasing eGDR. Multivariable analysis showed hazard ratios (HRs) for ${\mathrm{eGDR}}_{\text{WC}}$ and ${\mathrm{eGDR}}_{\text{BMI}}$ of 1.152 (95% confidence interval [CI] 1.088–1.219; *p *< 0.001) and 0.998 (95% CI 0.936–1.064; *p* = 0.957), respectively. Addition of ${\mathrm{eGDR}}_{\text{WC}}$ to a model that included currently recognized cardiovascular risk factors markedly enhanced its predictive power compared with the baseline model (Harrell’s C-index, ${\mathrm{eGDR}}_{\text{WC}}$ versus Baseline model, 0.778 versus 0.768, *p* = 0.003; continuous net reclassification improvement (continuous-NRI) of 0.125, *p *< 0.001; integrated discrimination improvement (IDI) of 0.016, *p *< 0.001).

**Conclusions:**

Low eGDR independently predicts low survival of NSTE-ACS cases who underwent PCI.

## 1. Introduction

Nowadays, cardiovascular disease (CVD) causes about one-third of deaths worldwide, with the morbidity and deaths related to CVD, especially coronary artery disease (CAD), increasing year by year. In addition, aging is exacerbating this trend [1, 2]. Therefore, many clinical researchers are committed to exploring residual risk factors in CVD cases, discovering novel targets for intervention and formulating individualized and precise treatment plans [3, 4, 5]. Considered a critical risk factor for CAD, type 2 diabetes mellitus (T2DM) is also rising in terms of prevalence [1, 6]. Therefore, the application value of diabetes-related risk factors and assessment indicators in the pathogenesis and prognosis of CVD attracts more and more attention [7, 8, 9, 10, 11].

The hyperinsulinemic-euglycemic clamp is the gold standard for assessing insulin resistance (IR), but its extensive clinical application is limited due to high cost, time-consumption and invasiveness. In 2000, estimated glucose disposal rate (eGDR) was developed to evaluate insulin sensitivity in T1DM patients and the results were verified with the hyperinsulinemic-euglycemic clamp [12, 13]. eGDR was originally calculated based on waist-to-hip ratio (WHR), hypertension and glycosylated hemoglobin (HbA1c). However, researchers have found that using waist circumference (WC) and body mass index (BMI) instead of WHR to calculate eGDR yielded the same results [12, 14]. Nonetheless, higher eGDR indicates greater insulin sensitivity, and lower eGDR reflects stronger IR [15].

Recently, a study confirmed that lower eGDR levels have associations with higher risk of stroke and death [16]. Such associations were independent of other stroke and mortality risk factors. More importantly, in T2DM cases administered coronary artery bypass grafting (CABG), low eGDR was linked to enhanced risk of all-cause mortality, suggesting eGDR might constitute a critical risk factor for T2DM with ischemic heart disease [17]. However, the prognostic potential of eGDR for CAD patients undergoing percutaneous coronary intervention (PCI) is undefined. Therefore, the current work aimed to evaluate the prognostic capability of eGDR for non-ST-elevation acute coronary syndrome (NSTE-ACS) upon PCI treatment.

## 2. Materials and Methods

### 2.1 Study Population

**Fig. 1.**
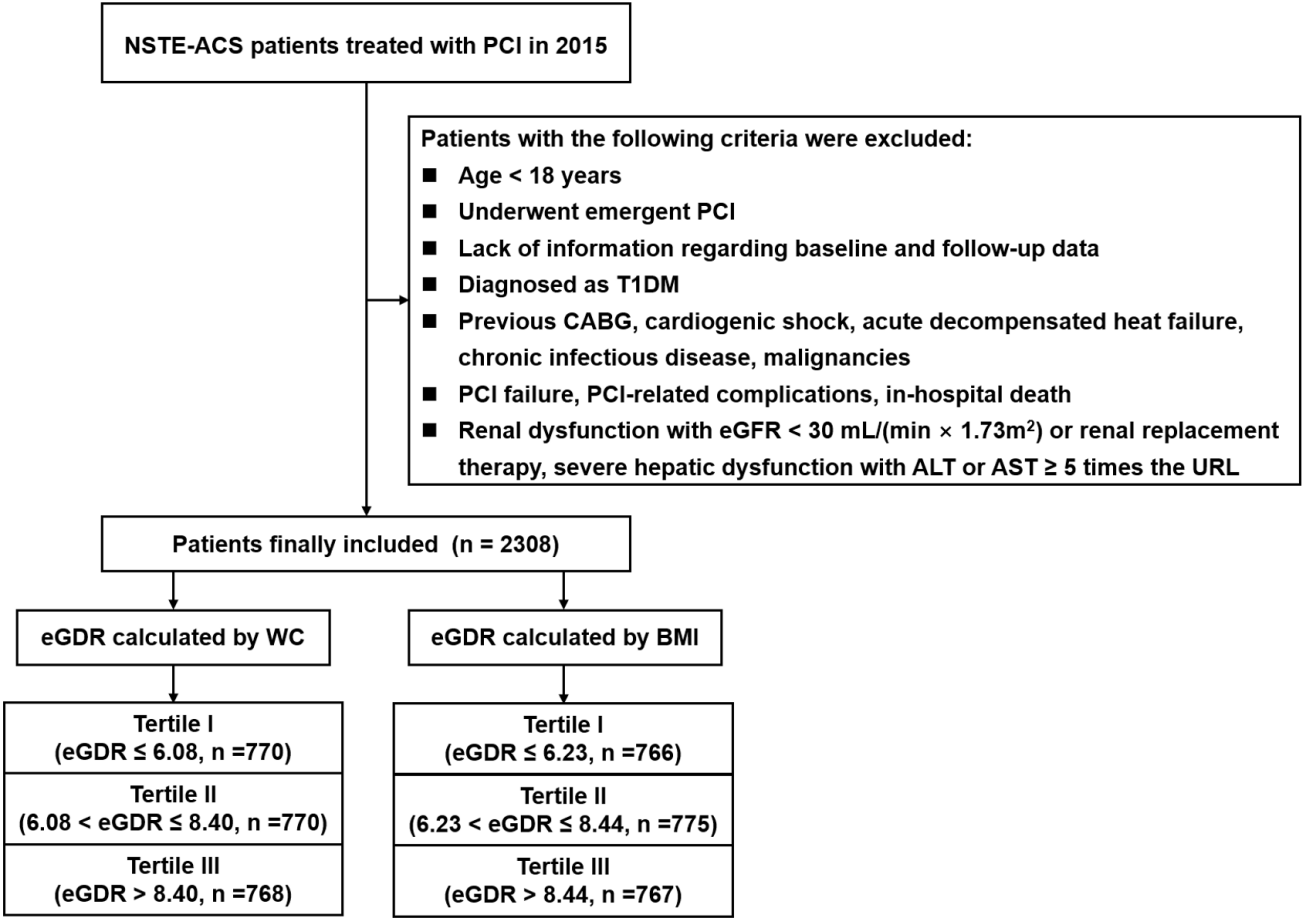
**Study flowchart**. NSTE-ACS, non-ST-segment elevation acute coronary syndrome; PCI, percutaneous coronary intervention; T1DM, Type 1 Diabetes mellitus; CABG, coronary artery bypass grafting; eGFR, estimated glomerular filtration rate; ALT, alanine transaminase; AST, aspartate transaminase; URL, upper reference limit; eGDR, estimated glucose disposal rate; WC, waist circumference; BMI, body mass index.

The present single-center, observational study enrolled NSTE-ACS patients undergoing PCI in Beijing Anzhen Hospital, China, between January 2015 and December 2015. NSTE-ACS diagnosis included non-ST-segment elevation myocardial infarction [NSTEMI] and unstable angina [UA] [18]. Exclusion criteria were: (1) age <18 years; (2) emergency PCI; (3) no baseline and/or follow-up data; (4) diagnosis of T1DM; (5) previous CABG, cardiogenic shock, acute decompensated heart failure, chronic infection or malignancies; (6) failed PCI, presence of PCI complications and/or in-hospital death; and (7) kidney function impairment with an estimated glomerular filtration rate (eGFR) <30 mL/(min × 1.73 $m^{2}$) or renal replacement treatment due to severely impaired liver function with alanine and/or aspartate transaminase levels ≥5 times the respective upper limits of normal values. Finally, 2308 patients were included (Fig. 1). The study had approval from the Clinical Research Ethics Committee of Beijing Anhui Hospital, and was carried out in accordance with the Helsinki Declaration.

### 2.2 Data Collection and Definitions

Patients’ demographics were derived from the hospital’s medical information record system. Definitions and diagnostic criteria for hypertension, T2DM, dyslipidemia, stroke and peripheral arterial disease (PAD) were based on current relevant guidelines [19, 20, 21, 22, 23, 24]. The calculation formula for BMI was ${\mathrm{weight/height}}^{2}$ (in ${\mathrm{kg/m}}^{2}$). WC was the girth of the midpoint line between the lowest point of the rib and the upper border of the iliac crest. Blood samples were drawn in the morning of surgery after fasting for 8–12 hours. Standard laboratory tests of hematological and biochemical parameters were performed. Echocardiograms were evaluated by two ultrasound physicians. Procedures for coronary intervention followed currently available guidelines [25, 26, 27]. Data related to coronary lesion characteristics were examined by two or more cardiologists with extended experience. Synergy between PCI with taxus and cardiac surgery (SYNTAX) scores were calculated by standard formula (https://syntaxscore.org/).

In this study, eGDR (mg/kg/min) was assessed according to previously proposed formulae [12, 14, 28]: eGDR calculated by WC (${\mathrm{eGDR}}_{\text{WC}}$) = 21.16 – (0.09 × WC [cm]) – (3.41 × Hypertension) – (0.55 × HbA1c [%]); eGDR calculated by BMI (${\mathrm{eGDR}}_{\text{BMI}}$) = 19.02 – (0.22 × BMI [${\mathrm{kg/m}}^{2}$]) – (3.26 × Hypertension) – (0.61 × HbA1c [%]).

### 2.3 Follow-Up and Study Endpoint

Follow-up duration was 48 months post-discharge or until death. Major adverse cardio-cerebral events (MACCEs), comprising all-cause death, non-fatal myocardial infarction (MI), non-fatal ischemic stroke and ischemia-associated revascularization, constituted the primary endpoint. MI was reflected by specific cardiac enzyme amounts surpassing the corresponding upper limits of their normal ranges, accompanied by ischemic symptoms or electrocardiographic changes suggestive of ischemia [29]. Stroke was any ischemic cerebral infarction requiring hospitalization accompanied by overt neurological dysfunction, with lesions demonstrated on brain computed tomography (CT) or magnetic resonance (MR) images. Ischemia-related revascularization referred to the revascularization of target and/or non-target vessels resulting from repeated or chronic ischemia.

### 2.4 Statistical Analysis

All 2308 patients were assessed by the parameters ${\mathrm{eGDR}}_{\text{WC}}$ and ${\mathrm{eGDR}}_{\text{BMI}}$, and assigned to 3 groups based on the tertiles of ${\mathrm{eGDR}}_{\text{WC}}$ (Tertile I [eGDR ≤6.08], Tertile II [6.08 < eGDR ≤ 8.40] and Tertile III [eGDR >8.40]) and ${\mathrm{eGDR}}_{\text{BMI}}$ (Tertile I [eGDR ≤6.23], Tertile II [6.23 < eGDR ≤ 8.44] and Tertile III [eGDR >8.44]), respectively.

Normally distributed continuous data are mean ± standard deviation (SD), and were compared by one-way analysis of variance. Continuous data with a non-normal distribution were presented as median with 25th and 75th percentiles, and the Kruskal–Wallis H test was utilized for between-group comparisons. Categorical variables were presented as number and percentage, and compared by the Chi-square, continuity-adjusted chi-square and Fisher’s exact tests.

Kaplan-Meier curve analysis was carried out for describing the cumulative rates of MACCEs (primary study endpoint) at different levels of eGDR, and between-group comparisons utilized the log-rank test. Univariate Cox regression analysis was utilized for initially identifying potential risk factors for MACCEs. Variables identified as potential risk factors for the primary endpoint in univariate analysis (*p *< 0.05) or considered potentially relevant clinically were further examined in 3 multivariate models, excluding those with possible collinearity. eGDR was assessed as both a nominal variable and a continuous variable. The hazard ratio (HR) and 95% confidence interval (CI) were determined for each parameter. In multivariable Cox proportional hazards analysis, 3 models with the following adjustments were built for assessing the predictive value of eGDR for NSTE-ACS: Model 1, age, sex, diabetes, hyperlipidemia, and previous MI, PCI and stroke; Model 2, Model 1 parameters plus triglyceride (TG), total cholesterol (TC), high-density lipoprotein cholesterol (HDL-C), eGFR, fasting blood glucose (FBG), and left ventricular ejection fraction (LVEF), and angiotensin-converting enzyme inhibitor (ACEI) or angiotensin receptor blocker (ARB), oral hypoglycemic agent (OHA) and insulin use at discharge; Model 3, Model 2 parameters plus left main artery (LM) lesion, multivessel lesion, in-stent restenosis, chronic total occlusion (CTO) lesion, SYNTAX score, LM lesion treatment, left circumflex artery (LCX) treatment, right coronary artery (RCA) treatment, complete revascularization and number of drug-eluting stents (DES) used.

On the basis of Model 3, the eGDR dose-response of the primary endpoint was represented by a restrictive cubic spline curve. The likelihood ratio test was carried out to examine the nonlinearity. Subgroup analyses stratified by sex, age, smoking history, hyperlipidemia, diabetes, OHA at admission and insulin at admission, with Model 3 adjustment, were performed to determine eGDR’s consistency in predicting MACCEs.

The incremental effects of eGDR on the predictive potential of currently recognized CVD risk factors for MACCEs were illustrated by the Harrell’s C-index, net reclassification improvement (NRI) and integrated discrimination improvement (IDI).

Statistical analysis was carried out with SPSS v26.0 (IBM Corp., Chicago, IL, USA) and R statistical software v3.6.3 (R Foundation for Statistical Computing, Vienna, Austria). Two-tailed *p *< 0.05 indicated statistical significance.

## 3. Results

### 3.1 Baseline Patient Features

A total of 2308 patients aged 60.09 ± 8.96 years were enrolled, with a male ratio of 71.8% (n = 1658). According to the tertiles of ${\mathrm{eGDR}}_{\text{WC}}$ and ${\mathrm{eGDR}}_{\text{BMI}}$, these patients were separated into Tertile I, Tertile II and Tertile III subgroups, respectively. Demographic, clinical and laboratory data, and details of drug and interventional therapies in the three subgroups of ${\mathrm{eGDR}}_{\text{WC}}$ and ${\mathrm{eGDR}}_{\text{BMI}}$ are presented in Table 1 and **Supplementary Table 1**.

**Table 1.** **Baseline characteristics of the study population in three groups of ${\mathrm{eGDR}}_{\text{𝐖𝐂}}$**.

			Total population (n = 2308)	Tertile I (n = 770) (eGDR ≤6.08)	Tertile II (n = 770) (6.08< eGDR ≤8.40)	Tertile III (n = 768) (eGDR >8.40)	*p* value
Age, years			60.09 ± 8.96	59.73 ± 8.71	61.15 ± 8.77	59.40 ± 9.31	<0.001
Sex, male, n (%)			1658 (71.8)	582 (75.6)	502 (65.2)	574 (74.7)	<0.001
BMI, ${\mathrm{kg/m}}^{2}$			26.09 ± 3.20	28.45 ± 2.81	25.18 ± 2.66	24.63 ± 2.68	<0.001
WC, cm			91.42 ± 12.38	101.46 ± 9.51	87.07 ± 10.43	85.71 ± 10.39	<0.001
Heart rate, bpm			69.67 ± 10.13	70.75 ± 10.66	69.51 ± 9.93	68.77 ± 9.69	0.001
SBP, mmHg			130.27 ± 16.45	133.25 ± 17.16	131.92 ± 16.49	125.62 ± 14.58	<0.001
DBP, mmHg			76.99 ± 9.77	78.88 ± 10.35	76.99 ± 9.61	75.08 ± 8.92	<0.001
Smoking history, n (%)			1309 (56.7)	461 (59.9)	400 (51.9)	448 (58.3)	0.004
Drinking history, n (%)			536 (23.2)	190 (24.7)	164 (21.3)	182 (23.7)	0.272
Family history of CAD, n (%)			236 (10.2)	73 (9.5)	79 (10.3)	84 (10.9)	0.641
Medical history, n (%)							
Diabetes			798 (34.6)	440 (57.1)	241 (31.3)	117 (15.2)	<0.001
	Hypertension		1436 (62.2)	759 (98.6)	641 (83.2)	36 (4.7)	<0.001
	Hyperlipidemia		1986 (86.0)	699 (90.8)	642 (83.4)	645 (84.0)	<0.001
	Previous MI		484 (21.0)	166 (21.6)	152 (19.7)	166 (21.6)	0.590
	Previous PCI		382 (16.6)	151 (19.6)	120 (15.6)	111 (14.5)	0.017
	Previous stroke		264 (11.4)	112 (14.5)	101 (13.1)	51 (6.6)	<0.001
	Previous PAD		79 (3.4)	30 (3.9)	25 (3.2)	24 (3.1)	0.670
Clinical diagnosis, n (%)							0.103
	UA		1921 (83.2)	623 (80.9)	652 (84.7)	646 (84.1)	
	NSTEMI		387 (16.8)	147 (19.1)	118 (15.3)	122 (15.9)	
Laboratory examinations							
	TG, mmol/L		1.48 (1.05, 2.10)	1.67 (1.21, 2.37)	1.46 (1.00, 2.02)	1.33 (0.96, 1.92)	<0.001
	TC, mmol/L		4.03 (3.40, 4.72)	4.02 (3.39, 4.75)	4.01 (3.40, 4.69)	4.08 (3.44, 4.76)	0.413
	LDL-C, mmol/L		2.39 (1.89, 2.98)	2.39 (1.88, 3.00)	2.35 (1.86, 2.92)	2.42 (1.92, 3.02)	0.147
	HDL-C, mmol/L		0.99 ± 0.23	0.93 ± 0.20	1.02 ± 0.25	1.01 ± 0.24	<0.011
	hs-CRP, mg/L		1.27 (0.58, 3.30)	1.76 (0.82, 4.23)	1.17 (0.52, 2.97)	1.00 (1.45, 2.64)	<0.001
	Creatinine, μmol/L		75.83 ± 16.52	77.40 ± 17.18	75.27 ± 16.57	74.83 ± 15.70	0.006
	eGFR, mL/(min × 1.73$m^{2}$)		93.57 ± 19.97	92.91 ± 20.95	92.28 ± 19.63	95.54 ± 19.15	0.002
	Uric acid, μmol/L		344.67 ± 80.75	353.63 ± 83.20	341.91 ± 79.36	338.46 ± 78.93	0.001
	FBG, mmol/L		6.13 ± 1.91	6.84 ± 2.35	6.03 ± 1.86	5.52 ± 1.04	<0.001
	HbA1c, %		6.27 ± 1.19	6.86 ± 1.37	6.15 ± 1.14	5.80 ± 0.72	<0.001
	LVEF, %		64.01 ± 6.72	63.69 ± 6.75	64.44 ± 6.30	63.90 ± 7.06	0.075
Medication at admission, n (%)							
	ACEI/ARB		511 (22.1)	258 (33.5)	215 (27.9)	38 (4.9)	<0.001
	DAPT		693 (30.0)	236 (30.6)	235 (30.5)	222 (28.9)	0.708
	Aspirin		1220 (52.9)	417 (54.2)	410 (53.2)	393 (51.2)	0.486
	P2Y12 inhibitors		738 (32.0)	245 (31.8)	251 (32.6)	242 (31.5)	0.895
	β-Blocker		505 (21.9)	183 (23.8)	187 (24.3)	135 (17.6)	0.002
	Statins		707 (30.6)	229 (29.7)	233 (30.3)	245 (31.9)	0.631
	OHA		413 (17.9)	237 (30.8)	125 (16.2)	51 (6.6)	<0.001
	Insulin		225 (9.7)	136 (17.7)	58 (7.5)	31 (4.0)	<0.001
Medication at discharge, n (%)							
	ACEI/ARB		1602 (69.4)	750 (97.4)	658 (85.5)	194 (25.3)	<0.001
	DAPT		2306 (99.9)	769 (99.9)	769 (99.9)	768 (100.0)	0.607
	Aspirin		2307 (100.0)	769 (99.9)	770 (100.0)	768 (100.0)	0.368
	P2Y12 inhibitors		2308 (100.0)	770 (100.0)	770 (100.0)	768 (100.0)	-
	β-Blocker		2095 (90.8)	707 (91.8)	711 (92.3)	677 (88.2)	0.008
	Statins		2256 (97.7)	752 (97.7)	755 (98.1)	749 (97.5)	0.771
	OHA		409 (17.7)	233 (30.3)	125 (16.2)	51 (6.6)	<0.001
	Insulin		217 (9.4)	128 (16.6)	58 (7.5)	31 (4.0)	<0.001
Angiographic data, n (%)							
	LM lesion		103 (4.5)	39 (5.1)	31 (4.0)	33 (4.3)	0.592
	Multi-vessel lesion		1536 (66.6)	585 (76.0)	511 (66.4)	440 (57.3)	<0.001
	In-stent restenosis		125 (5.4)	56 (7.3)	33 (4.3)	36 (4.7)	0.019
	Chronic total occlusion lesion		299 (13.0)	111 (14.4)	98 (12.7)	90 (11.7)	0.282
	SYNTAX score		10.61 ± 5.45	11.63 ± 5.66	10.41 ± 5.27	9.80 ± 5.27	<0.001
Procedural information							
	Target vessel territory, n (%)						
		LM	60 (2.6)	22 (2.9)	17 (2.2)	21 (2.7)	0.696
		LAD	1506 (65.3)	481 (62.5)	508 (66.0)	517 (67.3)	0.119
		LCX	804 (34.8)	301 (39.1)	272 (35.3)	231 (30.1)	0.001
		RCA	978 (42.2)	373 (48.4)	315 (40.9)	290 (37.8)	<0.001
	Complete revascularization, n (%)		1363 (59.1)	404 (52.5)	465 (60.4)	494 (64.3)	<0.001
	Number of DES		2.00 (1.00, 3.00)	2.00 (1.00, 3.00)	2.00 (1.00, 3.00)	1.00 (1.00, 2.00)	0.004

${\mathrm{eGDR}}_{\text{WC}}$, estimated glucose disposal rate calculated by waist circumference; eGDR, estimated glucose disposal rate; BMI, body mass index; WC, waist circumference; SBP, systolic blood pressure; DBP, diastolic blood pressure; CAD, coronary artery disease; MI, myocardial infarction; PCI, percutaneous coronary intervention; PAD, peripheral artery disease; UA, unstable angina; NSTEMI, non-ST-segment elevation myocardial infarction; TG, triglyceride; TC, total cholesterol; LDL-C, low-density lipoprotein cholesterol; HDL-C, high-density lipoprotein cholesterol; hs-CRP, high-sensitivity C-reactive protein; eGFR, estimated glomerular filtration rate; FBG, fasting blood glucose; HbA1c, glycosylated hemoglobin A1c; LVEF, left ventricular ejection fraction; ACEI, angiotensin-converting enzyme inhibitor; ARB, angiotensin receptor blocker; DAPT, dual antiplatelet therapy; OHA, oral hypoglycemic agents; LM, left main artery; SYNTAX, synergy between PCI with taxus and cardiac surgery; LAD, left anterior descending artery; LCX, left circumflex artery; RCA, right coronary artery; DES, drug-eluting stent.

The Tertile II ${\mathrm{eGDR}}_{\text{WC}}$ group had higher mean age and lower proportion of males compared with the other two subgroups. BMI, WC, heart rate, and systolic (SBP) and diastolic (DBP) blood pressure, TG, high-sensitivity C-reactive protein (hs-CRP), creatinine, uric acid, FBG and HbA1c levels, as well as the proportions of patients with diabetes, hypertension, previous PCI and previous stroke increased with decreasing ${\mathrm{eGDR}}_{\text{WC}}$ levels. The Tertile I ${\mathrm{eGDR}}_{\text{WC}}$ group had the highest incidence rates of smoking history and hyperlipidemia. HDL-C and eGFR were significantly different among the three groups. Regarding medications at admission and discharge, ACEI/ARB, OHA and insulin use increased with decreasing ${\mathrm{eGDR}}_{\text{WC}}$, and β-Blockers were primarily prescribed in the Tertile II ${\mathrm{eGDR}}_{\text{WC}}$ group. Regarding coronary angiography and PCI findings, SYNTAX score, the incidence of multi-vessel lesion, and LCX and RCA treatments showed an upward trend with decreasing ${\mathrm{eGDR}}_{\text{WC}}$, while complete revascularization showed a downward trend. In-stent restenosis and the number of DES showed significant differences among the three groups.

### 3.2 Incidence of MACCEs

During follow-up (mean follow-up time, 41.06 months), 547 patients (23.7%) had MACCEs, comprising 36 (1.6%) all-cause death, 112 (4.9%) non-fatal MI, 45 (1.9%) non-fatal ischemic stroke and 354 (15.3%) ischemia-induced revascularization. The rates of MACCEs (*p *< 0.001), non-fatal MI (*p* = 0.025), non-fatal ischemic stroke (*p* = 0.001) and ischemia-induced revascularization (*p *< 0.001) increased with decreasing ${\mathrm{eGDR}}_{\text{WC}}$. However, all-cause mortality rates were comparable among all three groups (Table 2). The incidence rates of the primary endpoint and its various components based on the tertile of ${\mathrm{eGDR}}_{\text{BMI}}$ are shown in **Supplementary Table 2**.

**Table 2.** **Incidence of primary endpoint and each component according to the tertile of ${\mathrm{eGDR}}_{\text{𝐖𝐂}}$**.

	Total population (n = 2308)	Tertile I (n = 770) (eGDR ≤6.08)	Tertile II (n = 770) (6.08< eGDR ≤8.40)	Tertile III (n = 768) (eGDR >8.40)	*p* value
MACCE, n (%)	547 (23.7)	261 (33.9)	159 (20.6)	127 (16.5)	<0.001
All-cause death, n (%)	36 (1.6)	14 (1.8)	12 (1.6)	10 (1.3)	0.716
Non-fatal MI, n (%)	112 (4.9)	49 (6.4)	37 (4.8)	26 (3.4)	0.025
Non-fatal ischemic stroke, n (%)	45 (1.9)	26 (3.4)	14 (1.8)	5 (0.7)	0.001
Ischemia-driven revascularization, n (%)	354 (15.3)	172 (22.3)	96 (12.5)	86 (11.2)	<0.001

${\mathrm{eGDR}}_{\text{WC}}$, estimated glucose disposal rate calculated by waist circumference; eGDR, estimated glucose disposal rate; MACCE, major adverse cardio-cerebral events; MI, myocardial infarction.

### 3.3 Cumulative Incidence of MACCEs at Follow-Up

Kaplan-Meier curve analysis was utilized for assessing the cumulative incidence of MACCEs in the overall, diabetic and non-diabetic cohorts.

**Fig. 2.**
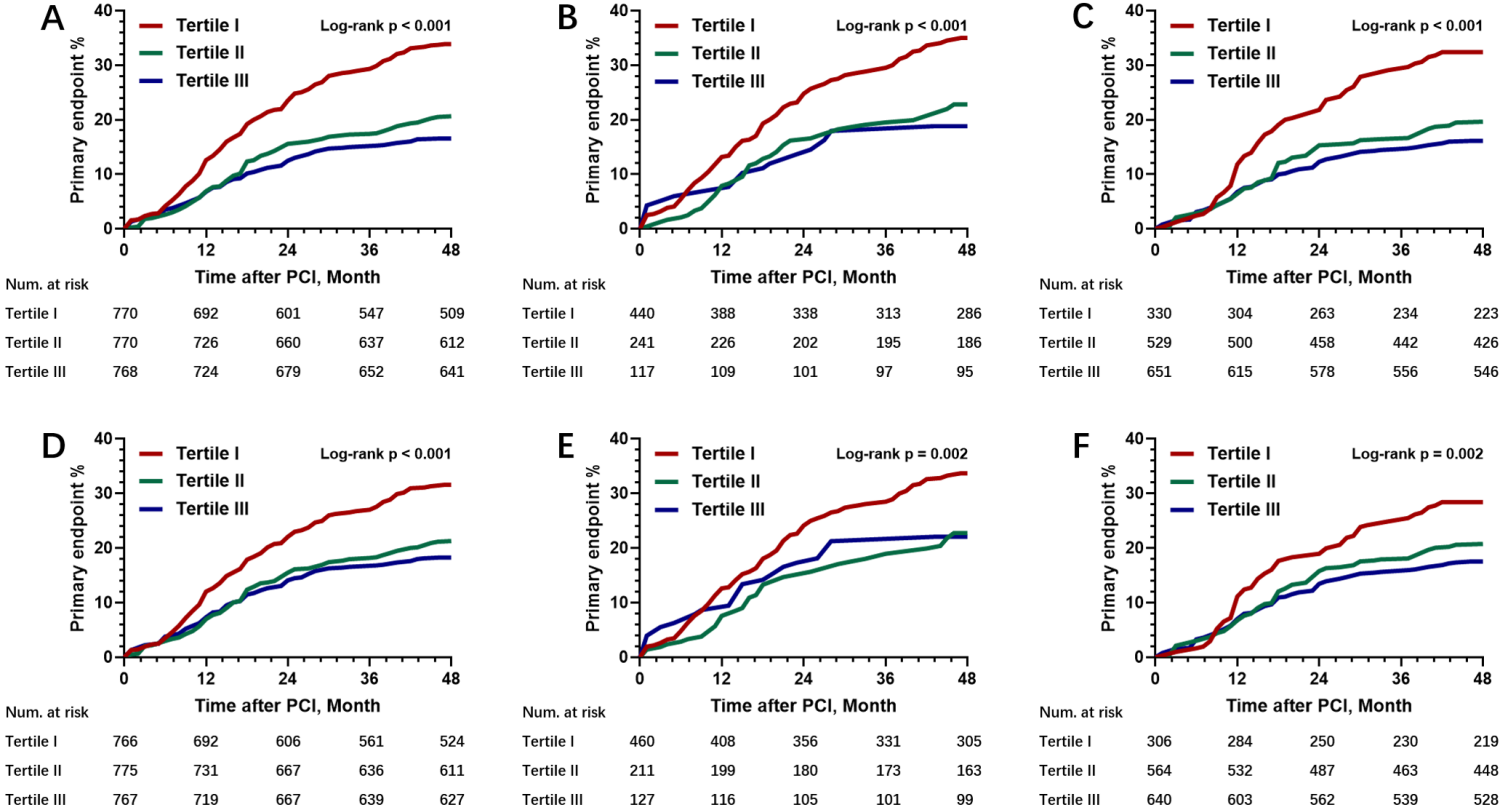
**Kaplan-Meier survival analysis based on the tertiles of eGDR**. (A) Kaplan-Meier survival curve analysis of the primary endpoint in the overall population for the three groups based on ${\mathrm{eGDR}}_{\text{WC}}$. (B) Kaplan-Meier survival curve analysis of the primary endpoint in diabetics for the three groups based on ${\mathrm{eGDR}}_{\text{WC}}$. (C) Kaplan-Meier survival curve analysis of the primary endpoint in non-diabetic cases for the three groups based on ${\mathrm{eGDR}}_{\text{WC}}$. (D) Kaplan-Meier survival curve analysis of the primary endpoint in the overall population for the three groups based on ${\mathrm{eGDR}}_{\text{BMI}}$. (E) Kaplan-Meier survival curve analysis of the primary endpoint for diabetics for the three groups based on ${\mathrm{eGDR}}_{\text{BMI}}$. (F) Kaplan-Meier survival curve analysis of the primary endpoint in non-diabetic cases for the three groups based on ${\mathrm{eGDR}}_{\text{BMI}}$. eGDR, estimated glucose disposal rate.

Statistically different cumulative incidence rates of MACCEs were found among the three ${\mathrm{eGDR}}_{\text{WC}}$ subgroups in the general, diabetic and non-diabetic cohorts (Fig. 2A–C, all log-rank *p *< 0.001). Similarly, the cumulative incidence rates of the primary endpoint were starkly different among the three ${\mathrm{eGDR}}_{\text{BMI}}$ subgroups in the general (Fig. 2D, log-rank *p *< 0.001), diabetic (Fig. 2E, log-rank *p* = 0.002) and non-diabetic (Fig. 2F, log-rank *p* = 0.002) cohorts.

### 3.4 Predictive Value of eGDR for MACCEs 

We built five multivariate models to examine the predictive potential of eGDR for the primary endpoint (shown in Methods). Univariable Cox proportional hazards analysis was performed to firstly determine the predictive factors for MACCEs (**Supplementary Table 3**). ${\mathrm{eGDR}}_{\text{WC}}$ had an independent prognostic value in three models, as both a nominal variable (Tertile I ${\mathrm{eGDR}}_{\text{WC}}$ versus Tertile III ${\mathrm{eGDR}}_{\text{WC}}$) and as a continuous variable (per 1-unit decrease in eGDR) (Table 3). However, as both a nominal variable (Tertile I ${\mathrm{eGDR}}_{\text{BMI}}$ versus Tertile III ${\mathrm{eGDR}}_{\text{BMI}}$) and a continuous variable (per 1-unit decrease in eGDR), ${\mathrm{eGDR}}_{\text{BMI}}$ showed an independent predictive potential only in Model 1, and not in Models 2–3 (**Supplementary Table 4**).

**Table 3.** **Predictive value of ${\mathrm{eGDR}}_{\text{𝐖𝐂}}$ for the risk of primary endpoint**.

	As nominal ${\mathrm{variate}}^{a}$				As continuous ${\mathrm{variate}}^{b}$	
	Tertile I HR (95% CI)	*p* value	Tertile II HR (95% CI)	*p* value	HR (95% CI)	*p* value
Unadjusted	2.247 (1.817–2.778)	<0.001	1.265 (1.002–1.597)	0.048	1.195 (1.149–1.242)	<0.001
Model 1	1.998 (1.592–2.509)	<0.001	1.137 (0.898–1.440)	0.286	1.181 (1.131–1.234)	<0.001
Model 2	1.794 (1.325–2.429)	<0.001	1.111 (0.837–1.474)	0.467	1.179 (1.115–1.246)	<0.001
Model 3	1.603 (1.190–2.159)	0.002	1.004 (0.761–1.326)	0.975	1.152 (1.088–1.219)	<0.001

Model 1: adjusted for age, sex, diabetes, hyperlipidemia, previous MI, previous PCI, previous stroke. Model 2: adjusted for variates in Model 1 and TG, TC, HDL-C, eGFR, FBG, LVEF, ACEI/ARB at discharge, OHA at discharge, insulin at discharge. Model 3: adjusted for variates in Model 2 and LM lesion, multi-vessel lesion, in-stent restenosis, chronic total occlusion lesion, SYNTAX score, LM treatment, LCX treatment, RCA treatment, complete revascularization, number of DES. ^a^The HR was evaluated regarding the Tertile III of eGDR as reference. ^b^The HR was evaluated by per 1-unit decrease of eGDR. ${\mathrm{eGDR}}_{\text{WC}}$, estimated glucose disposal rate calculated by waist circumference; HR, hazard ratio; CI, confidence interval.

### 3.5 ${\mathrm{eGDR}}_{\text{WC}}$ Dose-Response of MACCEs 

**Fig. 3.**
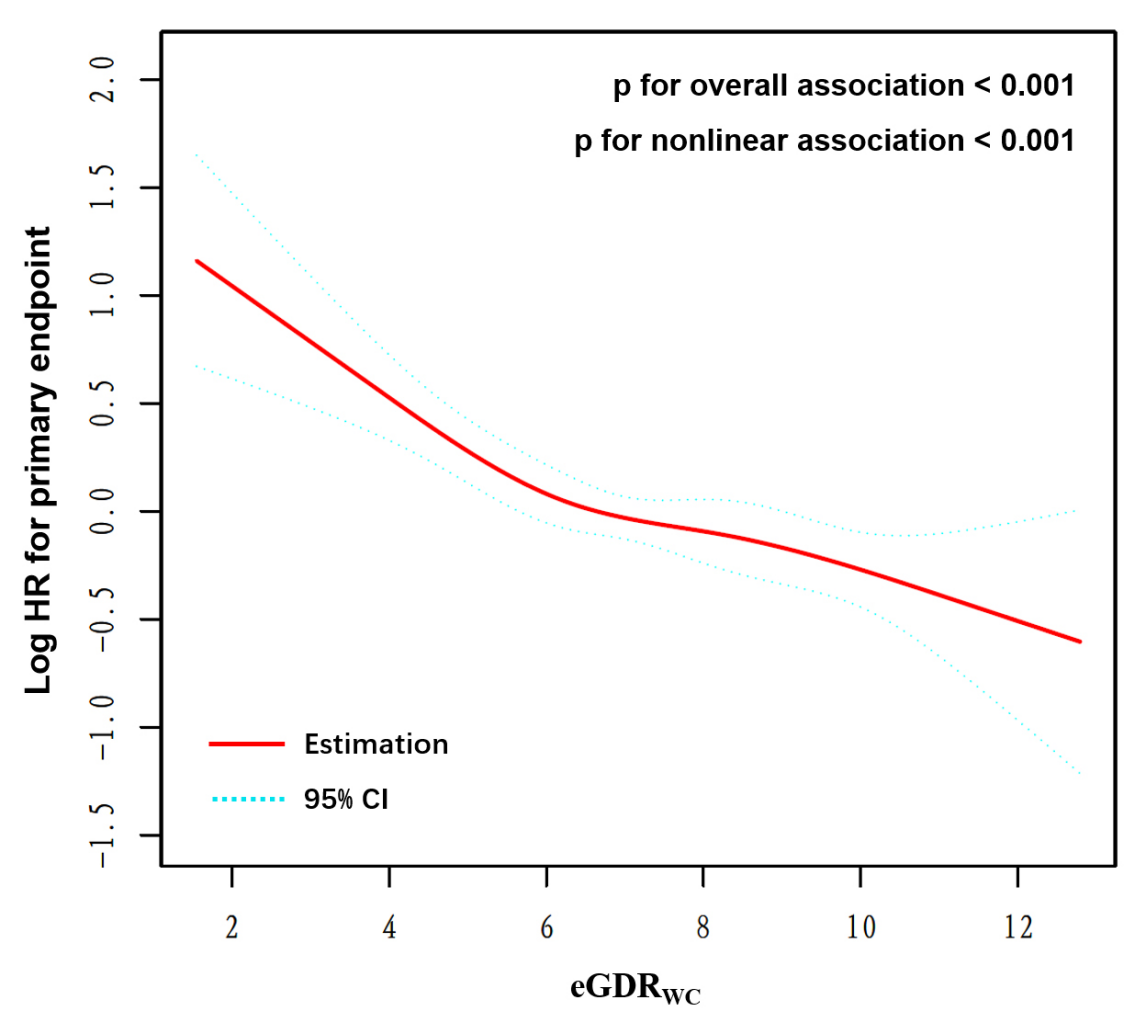
**Restricted cubic smoothing for the risk of the primary endpoint based on ${\mathrm{eGDR}}_{\text{𝐖𝐂}}$**. Adjustment was performed for Model 3. HR was assessed per 1-unit elevated of ${\mathrm{eGDR}}_{\text{WC}}$. ${\mathrm{eGDR}}_{\text{WC}}$, estimated glucose disposal rate calculated by waist circumference.

The ${\mathrm{eGDR}}_{\text{WC}}$ dose-response of the primary endpoint was examined by generating a restricted cubic spline curve (Fig. 3). The incidence of MACCEs decreased with increasing ${\mathrm{eGDR}}_{\text{WC}}$ (*p *< 0.001 for the overall association), suggesting a linear correlation of ${\mathrm{eGDR}}_{\text{WC}}$ with the risk of MACCEs. The above findings were verified by the nonlinear correlation test (*p *< 0.001 for nonlinear correlation).

### 3.6 Stratified Analysis of ${\mathrm{eGDR}}_{\text{WC}}$

**Fig. 4.**
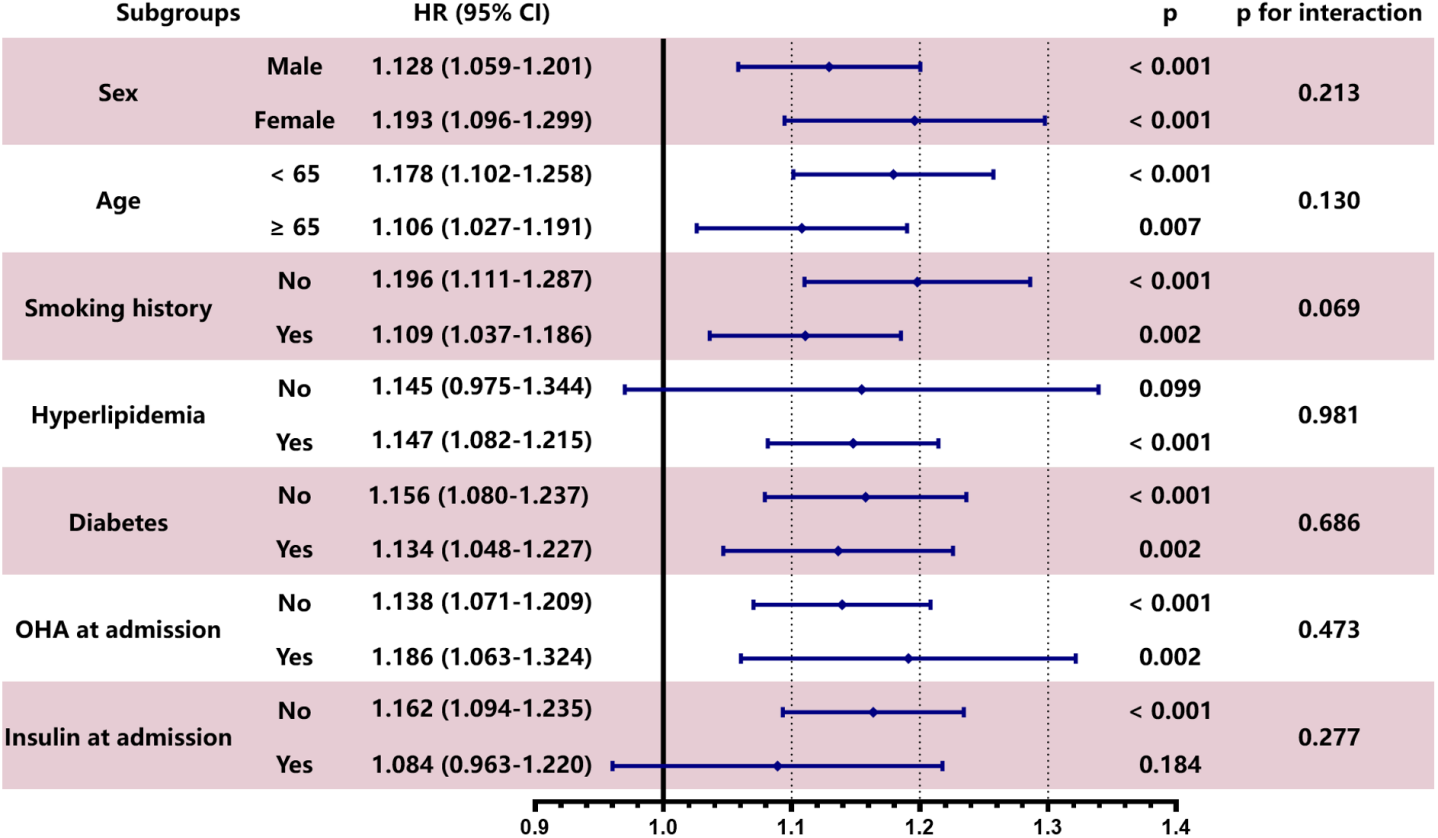
**Subgroup analysis evaluating the robustness of ${\mathrm{eGDR}}_{\text{𝐖𝐂}}$ in predicting the risk of the primary endpoint**. The analysis was adjusted for Model 3 except for variates applied for grouping. HR was evaluated by per 1-unit decrease of ${\mathrm{eGDR}}_{\text{WC}}$. OHA, oral hypoglycemic agents.

The predictive power of ${\mathrm{eGDR}}_{\text{WC}}$ for MACCEs did not differ among subgroups based on sex (male/female), age (<65/≥65 years), smoking history (no/yes), hyperlipidemia (no/yes), diabetes (no/yes), OHA at admission (no/yes) and insulin at admission (no/yes) (all *p* for interaction >0.05), further confirming the potential of ${\mathrm{eGDR}}_{\text{WC}}$ for predicting MACCEs (Fig. 4).

### 3.7 eGDR Enhances the Predictive Abilities of Other Parameters for MACCEs

Addition of ${\mathrm{eGDR}}_{\text{WC}}$ to the baseline model encompassing cardiovascular risk factors (sex, age, smoking history, hyperlipidemia, diabetes, MI history, stroke history, eGFR, NSTEMI, LVEF, SYNTAX score, complete revascularization and amount of DES; Harrell’s C-index: 0.768, *p* = 0.003) resulted in significantly improved predictive value (Harrell’s C-index: 0.778) and increased reclassification and discrimination abilities (continuous-NRI = 0.125, *p *< 0.001; IDI = 0.016, *p *< 0.001). However, adding ${\mathrm{eGDR}}_{\text{BMI}}$ did not starkly increase the baseline model’s predictive power (Harrell’s C-index: ${\mathrm{eGDR}}_{\text{BMI}}$, 0.769 versus Baseline model, 0.768, *p* = 0.198; continuous NRI: 0.061, *p* = 0.066; IDI: 0.002, *p* = 0.126) (Table 4).

**Table 4.** **Incremental effects of ${\mathrm{eGDR}}_{\text{𝐖𝐂}}$ and ${\mathrm{eGDR}}_{\text{𝐁𝐌𝐈}}$ on risk stratification for the primary endpoint beyond existing risk factors**.

	Harrell’s C-index			Continuous-NRI			IDI		
	Estimation	95% CI	*p* for comparison	Estimation	95% CI	*p* value	Estimation	95% CI	*p* value
Baseline model	0.768	0.750–0.786	-	-	-	-	-	-	-
${\mathrm{eGDR}}_{\text{WC}}$	0.778	0.760–0.796	0.003	0.125	0.067–0.176	<0.001	0.016	0.008–0.027	<0.001
${\mathrm{eGDR}}_{\text{BMI}}$	0.769	0.751–0.788	0.198	0.061	–0.009–0.109	0.066	0.002	0.000–0.006	0.126

NRI, net reclassification improvement; IDI, integrated discrimination improvement; CI, confidence interval; ${\mathrm{eGDR}}_{\text{WC}}$, estimated glucose disposal rate calculated by waist circumference; ${\mathrm{eGDR}}_{\text{BMI}}$, estimated glucose disposal rate calculated by body mass index.

## 4. Discussion

The current work mainly assessed eGDR’s predictive value for negative outcome in NSTE-ACS patients after PCI. The data revealed low eGDR was tightly correlated with high incidence of MACCEs. Reduction in eGDR represented a significant and independent predictive factor of adverse outcomes in the examined population. Furthermore, compared with eGDR calculated by BMI, eGDR determined by WC was more potent in predicting poor prognosis in NSTE-ACS individuals following PCI. Moreover, addition of eGDR improved the ability of the model incorporating currently recognized cardiovascular risk factors for predicting a negative prognosis.

eGDR was proposed for IR assessment in T1DM patients and validated by the hyperinsulinemic-euglycemic clamp, which ensures its accuracy to a certain extent. eGDR is a continuous variable and thus can be used as a dynamic measure to assess the effectiveness of a particular treatment. In T1DM patients, lower eGDR reflects greater risk, which promotes the occurrence of renal disease [30], peripheral vascular disease [31], CAD [32, 33] and death [34]. IR assessed by eGDR is considered the only factor consistently associated with all chronic complications of T1DM [35]. A cross-sectional study of T1DM patients found that individuals showing low eGDR had remarkably enhanced risk of CVD [36]. Additionally, eGDR effectively predicted survival outcomes tightly linked to all-cause mortality and cardiovascular mortality in T1DM cases [37]. Furthermore, similar to HbA1c, eGDR is also considered a reliable, clinically applicable marker for the assessment of T2DM and could be used to monitor the responses to specific treatments [14]. These results suggest eGDR can be used as an effective predictor of the occurrence and development of CVD. According to a nationwide observational study of 3256 individuals with T2DM who underwent CABG with a 3.1-year median follow-up, low eGDR was strongly correlated with an enhanced risk of all-cause mortality, independently of other cardiac vascular and metabolic risk factors [17]. The current results indicate eGDR better predicts long-term prognosis in patients undergoing revascularization. These patients often have severe coronary artery disease and poor control of risk factors, which requires more frequent prognostic evaluation. The characteristics of eGDR are only suitable for this requirement. Based on the above studies, this work also revealed consistent findings, further clarifying the predictive potential of eGDR reduction for adverse outcomes in NSTE-ACS individuals undergoing PCI. Multivariate and subgroup analyses suggested eGDR was a strong and stable predictor of prognosis in NSTE-ACS. This study also found that the predictive ability of ${\mathrm{eGDR}}_{\text{WC}}$ was more robust compared with that of ${\mathrm{eGDR}}_{\text{BMI}}$ in multivariate analysis. Moreover, the incremental effect of ${\mathrm{eGDR}}_{\text{WC}}$ on the predictive ability of CVD predictors for the primary endpoint was stronger in comparison with that of ${\mathrm{eGDR}}_{\text{BMI}}$. BMI is a currently recognized cardiovascular risk factor [38]. WC, which reflects visceral fat, is strongly correlated with IR and atherosclerotic cardiovascular disease (ASCVD) progression [39]. A meta-analysis of 82,864 individuals in nine UK national cohorts confirmed that WC, but not BMI, is associated with CVD-related mortality [40]. A study of 21,109 participants assessing the health status of American adults showed that WC has a higher discriminatory capability than BMI in predicting cardiac metabolic abnormalities, especially diabetes [41]. In the late period following PCI, WC showed biphasic U-shaped associations with cardiovascular outcomes and obesity [42]. Whether the prognostic value of ${\mathrm{eGDR}}_{\text{WC}}$ for NSTE-ACS patients undergoing PCI is greater than that of ${\mathrm{eGDR}}_{\text{BMI}}$ needs to be further determined in larger and better-designed studies. Homoeostasis model assessment of insulin resistance (HOMA-IR) represents a surrogate measure of IR based on fasting glucose and insulin levels. HOMA-IR has been widely used clinically due to its simplicity, low cost and effectiveness [43, 44], and has shown a high correlation with poor prognosis in CVD patients [45, 46, 47]. However, in clinical practice, fasting insulin levels are not routinely measured in diabetic patients, let alone non-diabetic patients. Moreover, the limited accuracy of insulin assessment techniques makes it hard to guarantee consistency across laboratories, particularly in case of low insulin amounts. Applying eGDR to evaluate the prognosis of CVD may remedy these deficiencies. A comparison of eGDR and HOMA-IR for predicting prognosis in CVD patients following PCI needs to be further performed. In the era of precision medicine, besides the DAPT score or bleeding risk score, there is no good tool to stratify and predict the risk of patients with NSTE-ACS and to select a personalized therapy based on individual risk. eGDR is easy to calculate, representing an effective tool for guiding the prevention and control of cardiovascular risk factors.

eGDR was calculated based on three factors, including hypertension, HbA1c and WC. Hypertension, with a well-known impact on ASCVD development and prognosis, is the most important component in the calculation formula [12]. HbA1c is a known predictor of CAD severity and early prognosis of stable angina pectoris [48]. In diabetics with successful DES implantation, HbA1c is highly correlated with enhanced risk of major adverse cardiovascular events [49]. In obesity, HbA1c is associated with both IR and underlying diseases such as hypertension, dyslipidemia, CVD and stroke [39, 50]. WC is the preferred index of the World Health Organization for the evaluation of central obesity. It shows a strong association with visceral fat content measured by CT, and is also linked to the incidence rates of cardiac death and non-fatal MI in patients undergoing PCI [42]. IR assessed by eGDR is independently correlated with carotid plaque burden in T1DM [51]. In addition, a study examining the correlations between eGDR and thrombotic biomarkers in T1DM patients showed eGDR is a suitable indicator of prothrombotic status, superior to BMI and insulin requirements [52].

This study had limitations. Firstly, given its single-center, retrospective, observational features, larger prospective multicenter trials are warranted to validate the present findings and improve the power of this analysis. Secondly, UA patients accounted for the majority of all NSTE-ACS cases in this study, so these results might not reflect the prognostic potential of eGDR in NSTEMI patients. Thirdly, this study did not compare the predictive powers of eGDR and HOMA-IR. Fourthly, the study population did not involve patients with emergent PCI and chronic coronary syndromes, and the findings need to be further validated in these populations. In addition, eGDR is a measure of IR in T1DM, and more evidence in the T2DM population is needed. Finally, only Chinese individuals were included, and the generalizability and stability of the above findings need to be verified in other ethnic groups.

## 5. Conclusions

In NSTE-ACS cases undergoing PCI, low eGDR is strongly linked to high MACCE incidence and constitutes an independent predictor of poor prognosis in NSTE-ACS. Incorporating eGDR greatly enhanced the predictive ability of currently accepted prognostic models. Furthermore, ${\mathrm{eGDR}}_{\text{WC}}$ has better predictive power than ${\mathrm{eGDR}}_{\text{BMI}}$ for NSTE-ACS individuals undergoing PCI.

## Data Availability

The datasets used in the current study are available from the corresponding author upon reasonable request.

## Abbreviations

CVD, cardiovascular disease; CAD, coronary artery disease; T2DM, type 2 diabetes mellitus; IR, insulin resistance; eGDR, estimated glucose disposal rate; T1DM, type 1 diabetes mellitus; WHR, waist-to-hip ratio; HbA1c, glycosylated hemoglobin; WC, waist circumference; BMI, body mass index; PCI, percutaneous coronary intervention; CABG, coronary artery bypass grafting; NSTE-ACS, non-ST-segment elevation acute coronary syndrome; NSTEMI, non-ST-segment elevation myocardial infarction; UA, unstable angina; eGFR, estimated glomerular filtration rate; PAD, peripheral arterial disease; SYNTAX, the synergy between PCI with taxus and cardiac surgery; ${\mathrm{eGDR}}_{\text{WC}}$, eGDR calculated by WC; ${\mathrm{eGDR}}_{\text{BMI}}$, eGDR calculated by BMI; MACCE, major adverse cardio-cerebral event; MI, myocardial infarction; CT, computed tomography; MR, magnetic resonance; SD, standard deviation; HR, hazard ratio; CI, confidence interval; TG, triglyceride; TC, total cholesterol; HDL-C, high-density lipoprotein cholesterol; FBG, fasting blood glucose; LVEF, left ventricular ejection fraction; ACEI, angiotensin-converting enzyme inhibitor; ARB, angiotensin receptor blocker; OHA, oral hypoglycemic agents; LM, left main artery; CTO, chronic total occlusion; LCX, left circumflex artery; RCA, right coronary artery; DES, drug-eluting stent; NRI, net reclassification improvement; IDI, integrated discrimination improvement; SBP, systolic blood pressure; DBP, diastolic blood pressure; hs-CRP, high-sensitivity C-reactive protein; ASCVD, atherosclerotic cardiovascular disease; HOMA-IR, homoeostasis model assessment of insulin resistance.

## Supplementary Material

Supplementary material associated with this article can be found, in the online version, at https://doi.org/10.31083/j.rcm2401002.
