# Supplementary material for: Prognostic Value of Estimated Glucose Disposal Rate in Patients with Non-ST-Segment Elevation Acute Coronary Syndromes Undergoing Percutaneous Coronary Intervention
Source: Rev Cardiovasc Med. 2023 Jan 3;24(1):2. doi: 10.31083/j.rcm2401002 (PMC11270396; doi:10.31083/j.rcm2401002)
Supplement: Supplementary file 1 [file 2153-8174-24-1-002-s1.zip › 2153-8174-24-1-002-s1.docx]

## Supplementary Table 1. Baseline characteristics of the study population in three groups of eGDR_BMI_.

|  | Total population  (n = 2308) | Tertile I (n = 766)  (eGDR ≤ 6.23) | Tertile II (n = 775)  (6.23 <eGDR ≤ 8.44) | Tertile III (n = 767)  (eGDR > 8.44) | *p* value |
| --- | --- | --- | --- | --- | --- |
| Age, years | 60.09 ± 8.96 | 60.18 ± 8.73 | 60.81 ± 8.70 | 59.28 ± 9.38 | 0.003 |
| Sex, male, n (%) | 1658 (71.8) | 511 (66.7) | 542 (66.9) | 605 (78.9) | <0.001 |
| BMI, kg/m^2^ | 26.09 ± 3.20 | 28.35 ± 2.91 | 24.98 ± 2.62 | 24.95 ± 2.78 | <0.001 |
| WC, cm | 91.42 ± 12.38 | 99.91 ± 11.03 | 86.91 ± 10.96 | 87.49 ± 10.50 | <0.001 |
| Heart rate, bpm | 69.67 ± 10.13 | 70.90 ± 10.59 | 69.40 ± 9.95 | 68.73 ± 9.74 | <0.001 |
| SBP, mmHg | 130.27 ± 16.45 | 133.93 ± 17.37 | 131.66 ± 16.18 | 125.21 ± 14.41 | <0.001 |
| DBP, mmHg | 76.99 ± 9.77 | 78.75 ± 10.54 | 77.27 ± 9.44 | 74.93 ± 8.88 | <0.001 |
| Smoking history, n (%) | 1309 (56.7) | 414 (54.0) | 426 (55.0) | 469 (61.1) | 0.009 |
| Drinking history, n (%) | 536 (23.2) | 167 (21.8) | 178 (23.0) | 191 (24.9) | 0.348 |
| Family history of CAD, n (%) | 236 (10.2) | 76 (9.9) | 79 (10.2) | 81 (10.6) | 0.918 |
| Medical history, n (%) |  |  |  |  |  |
| Diabetes | 798 (34.6) | 460 (60.1) | 211 (27.2) | 127 (16.6) | <0.001 |
| Hypertension | 1436 (62.2) | 761 (99.3) | 670 (86.5) | 5 (0.7) | <0.001 |
| Hyperlipidemia | 1986 (86.0) | 693 (90.5) | 647 (83.5) | 646 (84.2) | <0.001 |
| Previous MI | 484 (21.0) | 153 (20.0) | 158 (20.4) | 173 (22.6) | 0.410 |
| Previous PCI | 382 (16.6) | 141 (18.4) | 129 (16.6) | 112 (14.6) | 0.134 |
| Previous stroke | 264 (11.4) | 105 (13.7) | 109 (14.1) | 50 (6.5) | <0.001 |
| Previous PAD | 79 (3.4) | 27 (3.5) | 27 (3.5) | 25 (3.3) | 0.954 |
| Clinical diagnosis, n (%) |  |  |  |  | 0.267 |
| UA | 1921 (83.2) | 624 (81.5) | 650 (83.9) | 647 (84.4) |  |
| NSTEMI | 387 (16.8) | 142 (18.5) | 125 (16.1) | 120 (15.6) |  |
| Laboratory examinations |  |  |  |  |  |
| TG, mmol/L | 1.48 (1.05, 2.10) | 1.71 (1.23, 2.46) | 1.42 (0.99, 1.97) | 1.35 (0.98, 1.93) | <0.001 |
| TC, mmol/L | 4.03 (3.40, 4.72) | 4.05 (3.43, 4.81) | 3.99 (3.38, 4.65) | 4.05 (3.42, 4.76) | 0.413 |
| LDL-C, mmol/L | 2.39 (1.89, 2.98) | 2.40 (1.89, 3.00) | 2.34 (1.85, 2.90) | 2.42 (1.92, 3.02) | 0.147 |
| HDL-C, mmol/L | 0.99 ± 0.23 | 0.94 ± 0.20 | 1.01 ± 0.25 | 1.00 ± 0.23 | <0.001 |
| hs-CRP, mg/L | 1.27 (0.58, 3.30) | 1.75 (0.84, 4.23) | 1.16 (0.49, 2.90) | 1.00 (0.46, 2.65) | <0.001 |
| Creatinine, μmol/L | 75.83 ± 16.52 | 75.70 ± 17.38 | 76.00 ± 16.69 | 75.80 ± 15.47 | 0.942 |
| eGFR, mL/(min × 1.73m^2^) | 93.57 ± 19.97 | 92.84 ± 20.98 | 92.70 ± 19.87 | 95.19 ± 19.93 | 0.018 |
| Uric acid, μmol/L | 344.67 ± 80.75 | 349.49 ± 85.06 | 342.14 ± 76.69 | 342.42 ± 80.22 | 0.144 |
| FBG, mmol/L | 6.13 ± 1.91 | 6.90 ± 2.29 | 5.94 ± 1.87 | 5.55 ± 1.12 | <0.001 |
| HbA1c, % | 6.27 ± 1.19 | 6.95 ± 1.33 | 6.05 ± 1.15 | 5.81 ± 0.68 | <0.001 |
| LVEF, % | 64.01 ± 6.72 | 63.85 ± 6.60 | 64.35 ± 6.50 | 63.83 ± 7.02 | 0.235 |
| Medication at admission, n (%) |  |  |  |  |  |
| ACEI/ARB | 511 (22.1) | 266 (34.7) | 215 (27.7) | 30 (3.9) | <0.001 |
| DAPT | 693 (30.0) | 226 (29.5) | 241 (31.1) | 226 (29.5) | 0.727 |
| Aspirin | 1220 (52.9) | 399 (52.1) | 424 (54.7) | 397 (51.8) | 0.445 |
| P2Y12 inhibitors | 738 (32.0) | 236 (30.8) | 255 (32.9) | 247 (32.2) | 0.669 |
| β-Blocker | 505 (21.9) | 176 (23.0) | 189 (24.4) | 140 (18.3) | 0.010 |
| Statins | 707 (30.6) | 219 (28.6) | 239 (30.8) | 249 (32.5) | 0.255 |
| OHA | 413 (17.9) | 253 (33.0) | 104 (13.4) | 55 (7.3) | <0.001 |
| Insulin | 225 (9.7) | 138 (18.0) | 56 (7.2) | 31 (4.0) | <0.001 |
| Medication at discharge, n (%) |  |  |  |  |  |
| ACEI/ARB | 1602 (69.4) | 750 (97.9) | 684 (88.3) | 168 (21.9) | <0.001 |
| DAPT | 2306 (99.9) | 765 (99.9) | 775 (100.0) | 766 (99.9) | 0.603 |
| Aspirin | 2307 (100.0) | 765 (99.9) | 775 (100.0) | 767 (100.0) | 0.365 |
| P2Y12 inhibitors | 2308 (100.0) | 766 (100.0) | 775 (100.0) | 767 (100.0) |  |
| β-Blocker | 2095 (90.8) | 704 (91.9) | 712 (91.9) | 679 (88.5) | 0.032 |
| Statins | 2256 (97.7) | 750 (97.9) | 757 (97.7) | 749 (97.7) | 0.932 |
| OHA | 409 (17.7) | 249 (32.5) | 104 (13.4) | 56 (7.3) | <0.001 |
| Insulin | 217 (9.4) | 130 (17.0) | 56 (7.2) | 31 (4.0) | <0.001 |
| Angiographic data, n (%) |  |  |  |  |  |
| LM lesion | 103 (4.5) | 40 (5.2) | 28 (3.6) | 35 (4.6) | 0.306 |
| Multi-vessel lesion | 1536 (66.6) | 595 (77.7) | 499 (64.4) | 442 (57.6) | <0.001 |
| In-stent restenosis | 125 (5.4) | 50 (6.5) | 37 (4.8) | 38 (5.0) | 0.248 |
| Chronic total occlusion lesion | 299 (13.0) | 106 (13.8) | 102 (13.2) | 91 (11.9) | 0.505 |
| SYNTAX score | 10.61 ± 5.45 | 11.79 ± 5.59 | 10.18 ± 5.29 | 9.86 ± 5.29 | <0.001 |
| Procedural information |  |  |  |  |  |
| Target vessel territory, n (%) |  |  |  |  |  |
| LM | 60 (2.6) | 19 (2.5) | 18 (2.3) | 23 (3.0) | 0.684 |
| LAD | 1506 (65.3) | 481 (62.8) | 511 (65.9) | 514 (67.0) | 0.197 |
| LCX | 804 (34.8) | 301 (39.3) | 277 (35.7) | 226 (29.5) | <0.001 |
| RCA | 978 (42.2) | 370 (48.3) | 318 (41.0) | 290 (37.8) | <0.001 |
| Complete revascularization, n (%) | 1363 (59.1) | 386 (50.4) | 491 (63.4) | 486 (63.4) | <0.001 |
| Number of DES | 2.00 (1.00, 3.00) | 2.00 (1.00, 3.00) | 2.00 (1.00, 3.00) | 1.00 (1.00, 2.00) | 0.004 |

*eGDR_BMI_* estimated glucose disposal rate calculated body mass index, *eGDR* estimated glucose disposal rate, *BMI* body mass index, *WC* waist circumference, *SBP* systolic blood pressure, *DBP* diastolic blood pressure, *CAD* coronary artery disease, *MI* myocardial infarction, *PCI* percutaneous coronary intervention, *PAD* peripheral artery disease, *UA* unstable angina, *NSTEMI* non-ST-segment elevation myocardial infarction, *TG* triglyceride, *TC* total cholesterol, *LDL-C* low-density lipoprotein cholesterol, *HDL-C* high-density lipoprotein cholesterol, *hs-CRP* high-sensitivity C-reactive protein, *eGFR* estimated glomerular filtration rate, *FBG* fasting blood glucose, *HbA1c* glycosylated hemoglobin A1c, *LVEF* left ventricular ejection fraction, *ACEI* angiotensin-converting enzyme inhibitor, *ARB* angiotensin receptor blocker, *DAPT* dual antiplatelet therapy, *OHA* oral hypoglycemic agents, *LM* left main artery, *SYNTAX* synergy between PCI with taxus and cardiac surgery, *LAD* left anterior descending artery, *LCX* left circumflex artery, *RCA* right coronary artery, *DES* drug-eluting stent.

## Supplementary Table 2. Incidence of primary endpoint and each component according to the tertile of eGDR_BMI_.

|  | Total population  (n = 2308) | Tertile I (n = 766)  (eGDR ≤ 6.23) | Tertile II (n = 775)  (6.23 <eGDR ≤ 8.44) | Tertile III (n = 767)  (eGDR > 8.44) | *p* value |
| --- | --- | --- | --- | --- | --- |
| MACCE, n (%) | 547 (23.7) | 242 (31.6) | 165 (21.3) | 140 (18.3) | <0.001 |
| All-cause death, n (%) | 36 (1.6) | 13 (1.7) | 13 (1.7) | 10 (1.3) | 0.782 |
| Non-fatal MI, n (%) | 112 (4.9) | 43 (5.6) | 39 (5.0) | 30 (3.9) | 0.288 |
| Non-fatal ischemic stroke, n (%) | 45 (1.9) | 26 (3.4) | 14 (1.8) | 5 (0.7) | <0.001 |
| Ischemia-driven revascularization, n (%) | 354 (15.3) | 160 (20.9) | 99 (12.8) | 95 (12.4) | <0.001 |

*eGDR_BMI_* estimated glucose disposal rate calculated by body mass index, *eGDR* estimated glucose disposal rate, *MACCE* major adverse cardio-cerebral events, *MI* myocardial infarction.

## Supplementary Table 3. Unadjusted Cox regression analysis investigating predictors of primary endpoint.

|  | Primary endpoint | | |
| --- | --- | --- | --- |
|  | HR | 95% CI | *p* value |
| Age, per 1 years | 1.220 | 1.019–1.461 | 0.030 |
| Gender, male as reference | 1.036 | 1.026–1.046 | <0.001 |
| BMI, per 1 kg/m^2^ | 1.031 | 1.004–1.058 | 0.023 |
| WC, per 1 cm | 1.034 | 1.028–1.041 | <0.001 |
| Heart rate, per 1 bpm | 1.007 | 0.999–1.015 | 0.089 |
| SBP, per 1 mmHg | 1.007 | 1.002–1.012 | 0.010 |
| DBP, per 1 mmHg | 1.000 | 0.991–1.008 | 0.914 |
| Smoking history | 0.927 | 0.783–1.097 | 0.378 |
| Drinking history | 0.853 | 0.694–1.049 | 0.132 |
| Family history of CAD | 1.047 | 0.798–1.375 | 0.738 |
| Diabetes | 1.448 | 1.222–1.716 | <0.001 |
| Hypertension | 1.269 | 1.062–1.517 | 0.009 |
| Hyperlipidemia | 2.341 | 1.684–3.255 | <0.001 |
| Previous MI | 2.273 | 1.905–2.711 | <0.001 |
| Previous PCI | 1.757 | 1.445–2.136 | <0.001 |
| Previous stroke | 1.926 | 1.551–2.390 | <0.001 |
| Previous PAD | 1.192 | 0.771–1.844 | 0.430 |
| TG, per 1 mmol/L | 1.546 | 1.433–1.668 | <0.001 |
| TC, per 1 mmol/L | 1.108 | 1.025–1.097 | 0.009 |
| LDL–C, per 1 mmol/L | 1.031 | 0.939–1.132 | 0.521 |
| HDL–C, per 1 mmol/L | 0.324 | 0.218–0.481 | <0.001 |
| hs–CRP, per 1 mg/L | 1.012 | 1.000–1.025 | 0.057 |
| Creatinine, per 1 μmol/L | 1.005 | 1.000–1.010 | 0.048 |
| eGFR, per 1 mL/(min × 1.73m^2^) | 0.991 | 0.987–0.996 | <0.001 |
| Uric acid, μmol/L | 1.000 | 0.999–1.001 | 0.727 |
| FBG, per 1 mmol/L | 1.116 | 1.083–1.149 | <0.001 |
| HbA1c, per 1% | 1.263 | 1.193–1.337 | <0.001 |
| LVEF, per 1% | 0.961 | 0.951–0.972 | <0.001 |
| ACEI/ARB at admission | 1.225 | 1.011–1.486 | 0.038 |
| DAPT at admission | 1.260 | 1.057–1.504 | 0.010 |
| Aspirin at admission | 1.200 | 1.013–1.421 | 0.035 |
| P2Y12 inhibitors at admission | 1.264 | 1.062–1.504 | 0.008 |
| Statins at admission | 1.066 | 0.891–1.276 | 0.485 |
| OHA at admission | 1.313 | 1.072–1.608 | 0.008 |
| Insulin at admission | 1.618 | 1.270–2.062 | <0.001 |
| ACEI/ARB at discharge | 1.666 | 1.360–2.042 | <0.001 |
| DAPT at discharge | 0.430 | 0.060–3.057 | 0.399 |
| Statins at discharge | 1.405 | 0.727–2.715 | 0.312 |
| OHA at discharge | 1.299 | 1.060–1.593 | 0.012 |
| Insulin at discharge | 1.633 | 1.278–2.087 | <0.001 |
| LM lesion | 2.493 | 1.854–3.353 | <0.001 |
| Multi–vessel lesion | 2.942 | 2.342–3.696 | <0.001 |
| In–stent restenosis | 2.382 | 1.810–3.134 | <0.001 |
| Chronic total occlusion lesion | 2.900 | 2.391–3.516 | <0.001 |
| SYNTAX score, per 1–point | 1.130 | 1.115–1.146 | <0.001 |
| LM treatment | 2.270 | 1.542–3.343 | <0.001 |
| LAD treatment | 1.112 | 0.929–1.330 | 0.246 |
| LCX treatment | 1.338 | 1.129–1.587 | 0.001 |
| RCA treatment | 1.601 | 1.353–1.893 | <0.001 |
| Complete revascularization | 0.585 | 0.495–0.692 | <0.001 |
| Number of DES, per 1 DES | 1.255 | 1.185–1.329 | <0.001 |

*BMI* body mass index, *WC* waist circumference, *SBP* systolic blood pressure, *DBP* diastolic blood pressure, *CAD* coronary artery disease, *MI* myocardial infarction, *PCI* percutaneous coronary intervention, *PAD* peripheral artery disease, *TG* triglyceride, *TC* total cholesterol, *LDL-C* low-density lipoprotein cholesterol, *HDL-C* high-density lipoprotein cholesterol, *hs-CRP* high-sensitivity C-reactive protein, *eGFR* estimated glomerular filtration rate, *FBG* fasting blood glucose, *HbA1c* glycosylated hemoglobin A1c, *LVEF* left ventricular ejection fraction, *ACEI* angiotensin-converting enzyme inhibitor, *ARB* angiotensin receptor blocker, *DAPT* dual antiplatelet therapy, *OHA* oral hypoglycemic agents, *LM* left main artery, *SYNTAX* synergy between PCI with taxus and cardiac surgery, *LAD* left anterior descending artery, *LCX* left circumflex artery, *RCA* right coronary artery, *DES* drug-eluting stent.

## Supplementary Table 4. Predictive value of eGDR_BMI_ for the risk of primary endpoint.

|  | As nominal variate^a^ | | | | As continuous variate^b^ | |
| --- | --- | --- | --- | --- | --- | --- |
|  | Tertile I HR (95% CI) | *p* value | Tertile II HR (95% CI) | *p* value | HR (95% CI) | *p* value |
| Unadjusted | 1.850 (1.503–2.278) | <0.001 | 1.172 (0.935–1.468) | 0.168 | 1.131 (1.083–1.180) | <0.001 |
| Model 1 | 1.570 (1.252–1.969) | <0.001 | 1.042 (0.830–1.309) | 0.721 | 1.085 (1.034–1.138) | 0.001 |
| Model 2 | 1.171 (0.860–1.596) | 0.316 | 0.904 (0.680–1.202) | 0.487 | 1.015 (0.952–1.083) | 0.639 |
| Model 3 | 1.047 (0.776–1.411) | 0.766 | 0.829 (0.629–1.091) | 0.180 | 0.998 (0.936–1.064) | 0.957 |

Model 1: adjusted for age, sex, diabetes, hyperlipidemia, previous MI, previous PCI, previous stroke.

Model 2: adjusted for variates in Model 1 and TG, TC, HDL-C, eGFR, FBG, LVEF, ACEI/ARB at discharge, OHA at discharge, insulin at discharge.

Model 3: adjusted for variates in Model 2 and LM lesion, multi-vessel lesion, in-stent restenosis, chronic total occlusion lesion, SYNTAX score, LM treatment, LCX treatment, RCA treatment, complete revascularization, number of DES.

^a^The HR was evaluated regarding the Tertile III of eGDR as reference.

^b^The HR was evaluated by per 1-unit decrease of eGDR.

*eGDR_BMI_* estimated glucose disposal rate calculated by body mass index, *HR* hazard ratio, *CI* confidence interval.
